# Estimability index for volume quantification of homogeneous spherical lesions in computed tomography

**DOI:** 10.1117/1.JMI.5.3.031404

**Published:** 2017-12-11

**Authors:** Ehsan Samei, Marthony Robins, Baiyu Chen, Greeshma Agasthya

**Affiliations:** a Duke University, Department of Radiology, Durham, North Carolina, United States; b Duke University, Medical Physics Graduate Program, Durham, North Carolina, United States; c Duke University Medical Center, Carl E. Ravin Advanced Imaging Laboratories, Durham, North Carolina, United States; d Duke University, Departments of Physics, Biomedical Engineering, and Electronical and Computer Engineering, Durham, North Carolina, United States

**Keywords:** quantitative imaging volumetry, computed tomography nodule volume quantification, precision, estimability index (*e*′), task transfer function, noise power spectrum, biomarker

## Abstract

Volume of lung nodules is an important biomarker, quantifiable from computed tomography (CT) images. The usefulness of volume quantification, however, depends on the precision of quantification. Experimental assessment of precision is time consuming. A mathematical estimability model was used to assess the quantification precision of CT nodule volumetry in terms of an index ($e^{\prime }$), incorporating image noise and resolution, nodule properties, and segmentation software. The noise and resolution were characterized in terms of noise power spectrum and task transfer function. The nodule properties and segmentation algorithm were modeled in terms of a task function and a template function, respectively. The $e^{\prime }$ values were benchmarked against experimentally acquired precision values from an anthropomorphic chest phantom across 54 acquisition protocols, 2 nodule sizes, and 2 volume segmentation softwares. $e^{\prime }$ exhibited correlation with experimental precision across nodule sizes and acquisition protocols but dependence on segmentation software. Compared to the assessment of empirical precision, which required ∼300  h to perform the segmentation, the $e^{\prime }$ method required ∼3  h from data collection to mathematical computation. A mathematical modeling of volume quantification provides efficient prediction of quantitative performance. It establishes a method to verify quantitative compliance and to optimize clinical protocols for chest CT volumetry.

## 1 Introduction

The high-axial-resolution images of multidetector computed tomography (CT) have enabled the quantification of lung nodule volume, which is an important biomarker for cancer diagnosis and treatment response monitoring.^1^ For example, the change of nodule volume over time, assessed from serial scans of the patient, can be employed to ascertain treatment response and to differentiate benign and malignant nodules.^2^ The usefulness of a volume quantification technique, however, depends on the precision of the quantification, which is the degree to which repeated quantifications of the same nodule under unchanged conditions yield the same outcome. To confidently assess nodule volume change, it is important to have a detailed knowledge of the quantification precision. Furthermore, since the precision might depend on acquisition parameters and nodule characteristics, the assessment needs to be performed across a wide range of conditions.

In recent years, a number of studies have been performed to assess the quantification precision experimentally.^1^^,^^3^^–^^6^ The experimental method is effective but extremely time consuming, as it involves multiple steps of case preparation, repeated scans, nodule segmentation, and statistical analysis. In addition, due to the complexity of CT acquisition and reconstruction parameters (dose, pitch, kernel, slice thickness, etc.), the experimental results are difficult to generalize.

To assess the quantification precision in an efficient and generalizable manner, a mathematical model named the estimability index ($e^{\prime }$) was developed by Richard and Samei.^7^ The $e^{\prime }$ predicted the quantification precision by modeling the nodule and image characteristics in Fourier domain. It was shown to be effective in predicting the theoretical quantification precision obtained via a maximum likelihood estimator. However, in that implementation of $e^{\prime }$, for each nodule characteristics, the model needed to be first trained with pre-known precision values, therefore, limiting the applicability of the model to a wide range of nodule characteristics. Furthermore, the model assumed ideal quantification software, which had *a priori* knowledge of the nodule’s physical properties (size, contrast, shape, and edge profile), and made optimal use of the knowledge to segment the nodule.

Inspired by this prior work and motivated by its limitations, this study aimed to extend the $e^{\prime }$ model by replacing the nodule-specific training process with a more efficient, physically based modeling process, and the imperfection of the quantification software was modeled in terms of the discrepancy between the actual nodule and the segmentation software’s expectation. Furthermore, the precision derived from the reformulated $e^{\prime }$ model was validated against empirical precision across 54 distinct acquisition protocols, 2 nodule sizes, and 2 segmentation softwares.

## 2 Methods

### 2.1 Estimability Index (e′)

A surrogate of volume quantification precision, the estimability index ($e^{\prime }$), was developed by mathematically modeling the three factors influencing the quantification performance: the image quality (noise and resolution), a set of nodule characteristics, and the volume segmentation software.

**Fig. 1:**
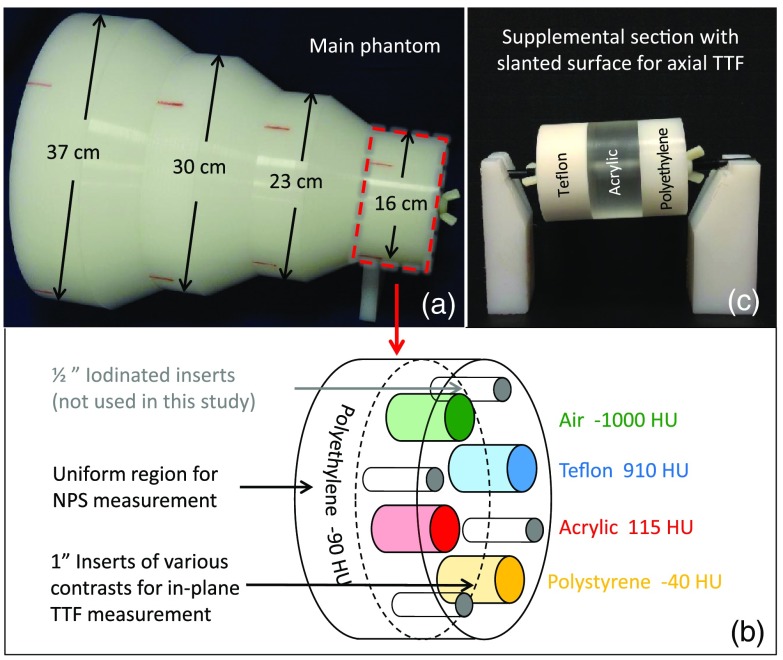
The 3-D noise and resolution properties of the imaging system are measured from Mercury Phantom in terms of NPS and TTF, respectively. (a) The phantom is composed of four cylindrical sections with three tapered sections in between. (b) Each cylindrical section is divided into two subsections for the measurements of NPS and in-plane TTF. (c) A supplemental section with slanted surfaces provides the measurement of TTF along axial direction.

The noise and resolution properties of the image were characterized in terms of noise power spectrum (NPS) and task transfer function (TTF), respectively. NPS is the square of the image noise (variance) as a function of spatial frequency, which describes both the magnitude and the texture of the noise. TTF is an extension of the modulation transfer function (MTF) to accommodate potential nonlinearity of iterative reconstruction (IR) algorithms by describing the image resolution as a function of object contrast and image noise.^8^^,^^9^ In this study, both NPS and TTF were measured for different contrast levels in three dimensions from a previously developed phantom, named the Mercury Phantom^10^ (Fig. 1). The three-dimensional (3-D) NPS was measured from the uniform region of the phantom; the in-plane TTF was measured from the rod inserts using an edge technique; and the axial TTF was measured from the interfaces between sections of the supplemental phantom, also using an edge technique. Both NPS and TTF measurements were described in detail in a previous study and have been validated for their accuracy and precision.^9^

The second factor of quantification, the physical properties of the nodule, was mathematically modeled in terms of task function, $W_{\text{task}}$. The task function was assumed as a 3-D rect function, differentiated by a Laplacian transform in Euclidean space, and subsequently Fourier transformed. If the nodule volume is defined as f(x,y,z), the below equation represents $W_{\text{task}}$ such that

$$W_{\text{task}}(f)=F[\nabla^{2}f(x,y,z)],$$ (1)

where

$$\nabla \;=(\frac{\partial f}{\partial x}+\frac{\partial f}{\partial y}+\frac{\partial f}{\partial z}).$$ (2)

**Fig. 2:**
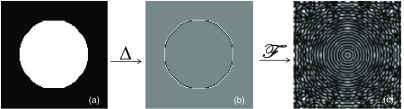
(a) A nodule is mathematically modeled. (b) The edge profile of the nodule is detected using a discrete Laplace operator. (c) The task function of the nodule is calculated as the Fourier transform of the edge profile. The nodule and its task function are 3-D but plotted in two dimensions for display purpose.

In that way, the task function is modeled as the 3-D Fourier transform of the nodule’s edge profile, containing information about the size, contrast, and edge profile of the nodule (Fig. 2). The magnitude of the task function is adjusted with a scale factor, such that the power of the function (the integral of the task function in frequency domain) equals the power of the nodule (the integral of the nodule in spatial domain).

The last component of the quantification precision, the nodule segmentation process, was modeled as a cross correlation between the nodule and a template, described in Fourier domain as a template function, $W_{\mathrm{temp}}$. If the template matches the nodule, the segmentation is optimized; if not, the segmentation is biased toward the template. The morphological processes employed by most commercial segmentation software favor spherical or lobular nodules.^11^ As a result, for most spherical nodules, the template matches the nodule well. However, spiculated nodules are penalized as they do not fit the spherical assumption of most segmentation algorithms.

Finally, inspired by the nonprewhitening matched filter model observer employed for detection tasks (detectability index),^12^ the $e^{\prime }$ was formulated as

$$\frac{1}{e^{\prime 2}}=\frac{∭\mathrm{NPS}\;\cdot {\mathrm{TTF}}^{2}\cdot |W_{\text{task}}|\cdot |W_{\text{temp}}|dudvdw}{{\left(∭{\mathrm{TTF}}^{2}\cdot |W_{\text{task}}|\cdot |W_{\text{temp}}|dudvdw\right)}^{2}},$$ (3)

where u,v, and w are the orthogonal spatial frequencies. $e^{\prime }$ modeled the quantification (segmentation) process as a cross correlation between the nodule and the template, which is equivalent to the product between the template function and the task function in Fourier domain. The numerator represents the fluctuation of the segmentation due to image noise and the mismatch between the nodule and the template, whereas the denominator normalizes it by the strength of the cross correlation, i.e., the similarity between the nodule and the template. Note that higher $e^{\prime }$ value represents larger fluctuation and therefore worse quantification precision. Higher noise (larger NPS), poorer resolution (lower TTF), or worse prior knowledge of nodule (mismatch between the template function and the task function) results in larger $e^{\prime }$.

### 2.2 Experimental Measurements of Quantification Precision

To verify our $e^{\prime }$ model, the gold standard of quantification precision was experimentally acquired in terms of percent repeatability coefficient (PRC), as described in detail in a previous study.^13^ PRC is the expected percentage difference between any two repeated quantifications of the same nodule, for 95% of cases.^13^^,^^14^ Smaller PRC, therefore, represents better quantification precision.

To calculate PRC, synthetic nodules (acrylic; 80 HU at 120 kVp) of 9.5 and 4.8 mm in diameters were embedded in an anthropomorphic chest phantom (LUNGMAN, KYOTO KAGAKU, Kyoto, Japan), attaching to lung vessel structures and pleura. The phantom was scanned repeatedly on a 64 slice CT scanner (Discovery CT750 HD, GE Healthcare, Waukesha, Wisconsin) with 54 distinct protocols, including 6 dose levels (100%, 75%, 50%, 25%, 10%, and 3%, with 100% dose corresponding to a ${\mathrm{CTDI}}_{\mathrm{vol}}$ of 7.5 mGy), 3 slice thicknesses (0.625, 1.25, and 2.5 mm), and 3 reconstruction algorithms [filtered back-projection (FBP), adaptive statistical iterative reconstruction (ASiR), and model-based iterative reconstruction (MBIR)]. Each protocol was repeated five times for precision calculation. Nodule volumes were quantified from the phantom images using two semiautomatic clinical segmentation software (software A: LungVCAR, GE Healthcare, Waukesha, Wisconsin, and software B: iNtuition, TeraRecon, Foster City, California). 108 PRC values were calculated from the quantified volumes, corresponding to the 54 acquisition protocols and the 2 nodule sizes.

### 2.3 Relating e′ to Experimental Precision

$e^{\prime }$ was then calculated for the same acquisition protocols and nodule sizes employed in the PRC calculation. First, a library of TTF and NPS was derived from the Mercury Phantom measurements, characterizing the resolution and noise properties of the operating space under a range of dose levels, reconstruction algorithms, and slice thicknesses. Specific TTF and NPS relevant to the measurement condition of PRC were then interpolated from the library according to the nodule contrast, the noise magnitude, the reconstruction algorithm, and the slice thickness of the chest phantom images. Because of IR algorithms exhibit different noise magnitudes in textured region and in uniform region,^13^^,^^15^ the noise magnitude used for interpolations was measured from the lung region. In that process, lung vessels were removed prior to the noise measurements by subtracting repeat scans. In total, 54 pairs of TTF and NPS were interpolated from the library to represent the resolution and noise properties of the 54 protocols used in PRC calculations. The task functions were modeled for the two types of nodules used for PRC calculations. The template functions were modeled as identical to the task functions since the nodules were perfectly spherical. Finally, $e^{\prime }$ was calculated across all protocols and nodule sizes and related to PRC to see how it may predict quantification precision. Relationships were established between $e^{\prime }$ and PRC, which were further employed to develop a process for $e^{\prime }$-based PRC prediction.

## 3 Results

### 3.1 Reconstructed Images

**Fig. 3:**
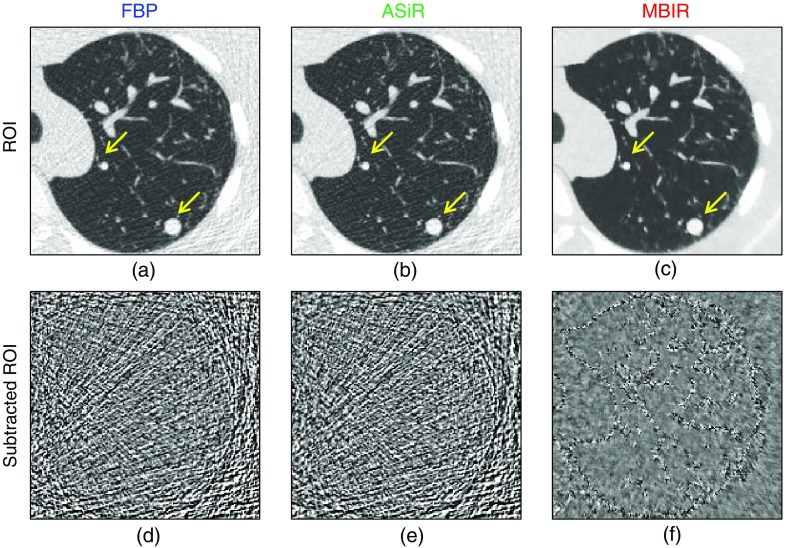
Regions of interest showing the anthropomorphic chest phantom (used for PRC calculation) reconstructed at 0.625-mm slice thickness and 3% dose with three algorithms: FBP, ASiR, and MBIR. (a)–(c) The nodules being quantified are highlighted with arrows and (d)–(f) subtracted regions of interest showing the noise only.

As a visual example of how acquisition parameters impact the image appearance and further quantification precision, images of the anthropomorphic chest phantom reconstructed with three algorithms are shown in Figs. 3(a)–3(c). The same images with noise only (structure removed by subtracting repeated scans) are shown in Figs. 3(d)–3(f). Both ASiR and MBIR showed noise reduction as compared to FBP. MBIR had more noise reduction in the relatively uniform areas of the images (e.g., soft tissue) than around lung vessels and nodules.

### 3.2 Task Transfer Function and Noise Power Spectrum

**Fig. 4:**
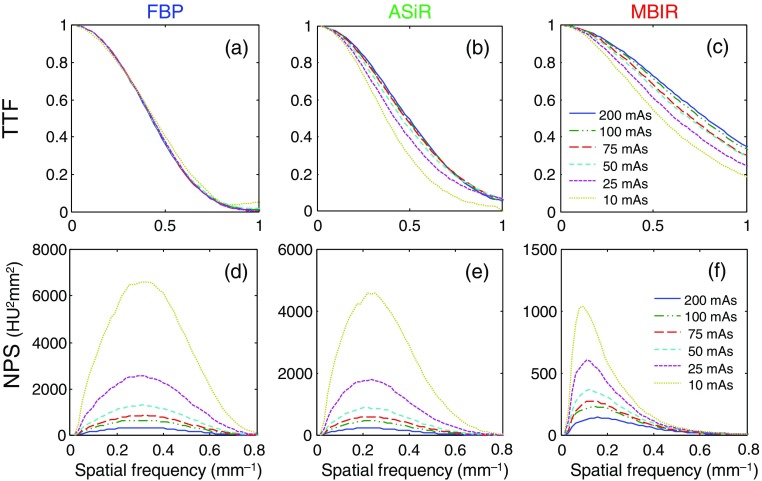
The TTF of three reconstruction algorithms (FBP, ASiR, and MBIR) at various dose levels. (a)–(c) The contrast level for TTF measurement is fixed at 1000 HU and (d)–(f) the NPS of the three reconstruction algorithms at various dose levels.

Figure 4 shows examples of TTF and NPS measured at various dose levels for all three reconstruction algorithms, with the slice thickness fixed at 1.25 mm. Although 3-D TTF and NPS were used for $e^{\prime }$ calculation, Fig. 4 only shows the in-plane radially averaged results for visualization and comparison purposes. Compared to FBP, the two IR algorithms demonstrate enhanced TTF, i.e., better resolution, but also a noise dependency, with higher dose (lower image noise) levels yielding better resolution. The two IR algorithms showed NPS curves with smaller magnitude but lower peak frequency, reflecting IR’s lower frequency noise feature, i.e., “waxier” appearance (as shown in Fig. 3).

### 3.3 e′ Results and Validation

**Fig. 5:**
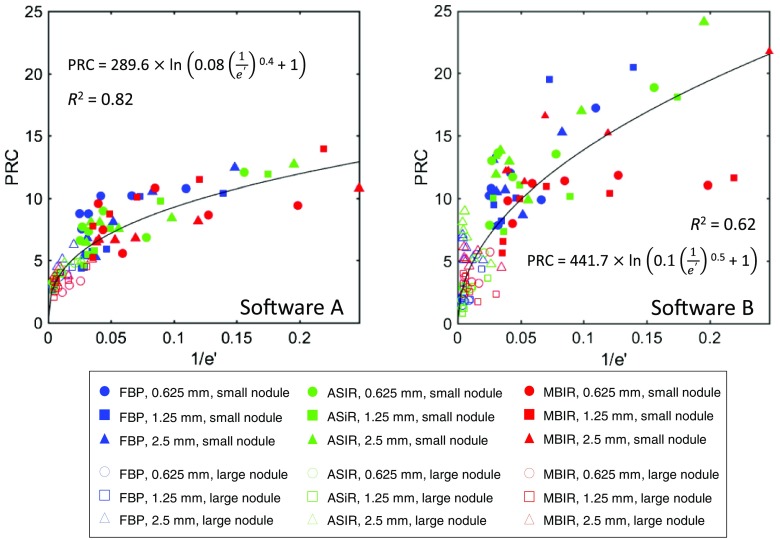
PRC verses $1/e^{\prime }$ across three reconstruction algorithms, three slice thicknesses, six dose levels, two nodule sizes, and two segmentation software algorithms.

Figure 5 shows the relationship between $e^{\prime }$ and PRC for 54 protocols, 2 nodule sizes, and 2 segmentation softwares. The relationships between $e^{\prime }$ and PRC exhibit variability primarily due to the nature of the PRC quantification.^13^ In previous studies, the relationship between $d^{\prime }$ and the area under the curve was shown to be an asymptotic one based on the error function that closely matches a logarithmic relationship.^16^^,^^17^ Similarly, these empirical data suggest that comparison between PRC and $1/e^{\prime }$ follows an analogous pattern. This general nonlinear relationship of the data was thus fitted to the form $\mathrm{PRC}=a\;\ln(b\cdot e^{\prime c}+1)$. In general, the relationships were found to be independent of nodule size, imaging dose, reconstruction algorithm, and slice thickness but dependent on the segmentation software, with software A showing a stronger correlation ($R^{2}=0.82$) than software B with ($R^{2}=0.62$).

### 3.4 Process for e′ Benchmarking

**Fig. 6:**
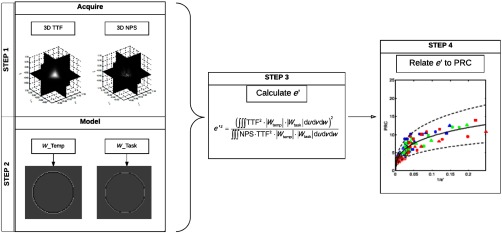
Flow chart summarizes the process for $e^{\prime }$-based assessment of PRC. Step 1: TTF and NPS that characterize the operating system are acquired. Step 2: $W_{\text{task}}$ and $W_{\text{temp}}$ are modeled according to the nodule size, shape, and contrast. Step 3: $e^{\prime }$ is calculated according to TTF, NPS, $W_{\text{task}}$, and $W_{\text{temp}}$. Step 4: $e^{\prime }$ is related to PRC using the relationship established in Fig. 5.

With the relationships established between $e^{\prime }$ and PRC, a process for $e^{\prime }$-based PRC assessment is summarized in Fig. 6. The process is based on four steps. In step 1, the image quality phantom is scanned several times to establish a library of TTF and NPS characterizing the resolution and noise properties of the operating space. A range of dose levels, slice thicknesses, and reconstruction algorithms are used to compute a library of TTF and NPS that characterizes the operating space. Step 2 models the task function and the template function and combines them with the library to calculate the PRC. In step 3, $e^{\prime }$ is calculated according to TTF, NPS, $W_{\text{task}}$, and $W_{\mathrm{temp}}$, before being related to PRC using the relationships established in Fig. 5.

The $e^{\prime }$-based PRC assessment boosted image processing efficiency considerably as compared to experimental-based PRC assessment. The experimental-based assessment in this study involved 30 scans (6 dose levels ×5 repeats) and 9 reconstructions per scan (3 reconstruction algorithms ×3 slice thicknesses). Furthermore, each dataset contained 35 nodules (21 9.5- and 14 4.8-mm nodules) to be semiautomatically segmented with 2 softwares, yielding a total of 18,900 segmentations. On the contrary, the calculation of $e^{\prime }$ involved only 6 scans (6 dose levels) and 9 reconstructions per scan (3 reconstruction algorithms ×3 slice thicknesses) to establish a library of NPS and TTF, and mathematically calculated $e^{\prime }$ for all nodule types and acquisition protocols.

## 4 Discussion

To quantify the development of lung nodule volumes with confidence, the precision of CT volume quantification needs to be assessed for a wide range of image acquisition and reconstruction parameters and nodule characteristics. The traditional experimental assessment of precision is time consuming. As a result, this study developed a mathematical model to predict quantification precision in terms of an estimability index ($e^{\prime }$). Results showed a reasonable correlation between $e^{\prime }$ and empirical precision, indicating $e^{\prime }$ as a general predictor of quantification precision across three reconstruction algorithms, three slice thicknesses, six dose levels, two nodule sizes, and two segmentation softwares.

As mentioned in Sec. 1, this $e^{\prime }$ model was an extension of a prior $e^{\prime }$ methodology.^7^ One limitation of that work was that it relied on pre-known precision values to train the task function for each individual nodule type. In this study, by introducing the task function based on the principle of volumetry (edge detection), not only was the computational burden of the training process eliminated but also the possible bias introduced by the training data was avoided. Another limitation of the prior work was that it did not explicitly model the segmentation software as an influencing factor of precision performance. In this study, the imperfection of clinical segmentation software was modeled in two regards: first, a nonprewhitening matched filter model instead of a prewhitening model was used to capture the impact of noise correlation on quantification precision;^12^ second, a template function was introduced to account for the possible mismatch between the nodule and the software’s expectation.

In clinical practice, methods other than volume quantification are also employed to assess the size of lung nodule, such as the bidimensional quantification suggested by World Health Organization (WHO)^18^ and the unidimensional quantification suggested by Response Evaluation Criteria in Solid Tumors (RECIST) working group.^19^ This study chose to focus on volume quantification because it has been shown to be more accurate and precise compared to the bi- and unidimensional quantifications, especially for complex lesion shapes.^20^ To reflect the 3-D nature of volume quantification, the $e^{\prime }$ was modeled in three dimensions. However, similar $e^{\prime }$ models in one and two dimensions may be derived to evaluate the performance of quantifications using WHO and RECIST methods, if needed.

To model the segmentation process, the NPS employed in $e^{\prime }$ calculation needs to reflect the noise property in the lung region around the vessels and nodules. The NPS in this study, however, was measured from the uniform region of the image quality phantom. While this is not a problem for FBP reconstruction that has a noise property independent of background structures, this poses a problem for IR that has a higher noise in the lung region than in the uniform region. To account for this, this study adjusted the NPS measured in the uniform region according to the noise magnitudes measured directly in the lung region, under the assumption that the presence of structures only changed the magnitude of the noise, not the frequency component of the noise. Note that the presented NPS is a radial average, which blurs peaks that might have more of directional presentations. With the adjustment, the correlation between $e^{\prime }$ and empirical precision became independent of the reconstruction algorithm. However, it would be more precise to measure the NPS directly from a structured region. Current efforts in our group are developing image quality phantom with anthropomorphic structures and corresponding NPS measurement technique.

Visual inspection of images in Fig. 3 indicates different noise and sharpness attributes. ASiR and MBIR images exhibit lower noise than FBP but also different noise texture with a more patchy appearance (associated with noise regularization in IRs). But they also exhibit a higher resolution as depicted in the corresponding TTF reported in Fig. 4. The TTF of MBIR, in particular, exhibits high values at high spatial frequencies, whereas its NPS has high values at lower frequencies. This differs from what is typically observed with FBP, where NPS patterns resemble that of the MTF. IRs, by dissociating noise from the resolution properties of the imaging system, can control the two independently. This phenomenon is one of the key features of IRs.

The relationship between $e^{\prime }$ and PRC was found to be independent of the image acquisitions parameters. However, a previous study has shown that the effect of pitch on the repeatability of lung nodule volume is not negligible.^21^ Pitch was not studied in this work; hence, the effect of pitch on the relationship between $e^{\prime }$ and PRC has to be studied in the future.

The correlations between $e^{\prime }$ and PRC were weaker for software B than for software A. This does not necessarily indicate that $e^{\prime }$ is worse at predicting the precision of quantifications performed with software B. This is because PRC, the gold standard for quantification precision, has its own uncertainty due to (1) the response stability unique to each segmentation tool and (2) the limited sample size when estimated with a challengingly large number of nodules (21 9.5 and 14 4.8-mm nodules).^13^ In this study, because software B is a generic segmentation software and did not handle images from a GE scanner as stably as the GE-developed software A, the uncertainty associated with PRC measurements is larger for software B than for software A. While accuracy was not the focus of the work, the data also exhibited that, on average, volume measurements made using software A had percent bias that were more favorable than values for software B. In terms of $e^{\prime }$ predictability across algorithms, however, it should be noted that the $e^{\prime }$ computation relies on an edge-based task function. This could potentially lead to different results for different algorithms based on the algorithmic design. This is a topic that is worth further exploration in terms of defining different task functions based on algorithmic properties, if known.

The relationships established between $e^{\prime }$ and PRC were generally independent of image acquisition parameters for two nodule sizes but depend on volume segmentation software. This indicated imperfect modeling of the segmentation software in $e^{\prime }$. One possible solution is to replace the current software-generic template function with a software-specific template function, which closely models the segmentation algorithm of each software and its impact on quantification precision. Another possible solution is to include an “internal noise” term, which models the inconsistency of the software during repeated segmentations of identical dataset, and counts it toward precision deterioration. The methodology described in this paper should also be expanded across a wide range of CT scanners. These remain as possible future extensions of the $e^{\prime }$ methodology.

## 5 Conclusion

A mathematical model based on an estimability index was developed to assess the quantification precision efficiently. The model was shown effective across three reconstruction algorithms, three slice thicknesses, six dose levels, two nodule sizes, and two segmentation softwares algorithms for spherical homogeneous lesions. An extension of this study to a larger range of variables can be employed to generalize optimization of CT protocols over a wide range of imaging conditions.
